# Mechanical Discontinuity and Mechanobiology at the Graft–Host Junction: Enhancing Functional Integration in Soft-Tissue Implantation

**DOI:** 10.3390/bioengineering13101106

**Published:** 2026-09-23

**Authors:** Zhonggang Feng, Tetsuro Uchida

**Affiliations:** 1 Graduate School of Science and Engineering, Yamagata University, Yonezawa 992-8510, Japan; 2 Second Department of Surgery, Faculty of Medicine, Yamagata University, Yamagata 990-9585, Japan

**Keywords:** soft-tissue implantation, graft–host junction, clinical tissue engineering, mechanical mismatch, mechanobiology, immune coupling, spatiotemporal mechanobiology

## Abstract

Tissue-engineered constructs (TECs) offer promising alternatives to autologous grafts and conventional artificial implants for the repair or replacement of damaged soft tissues. Nevertheless, their clinical translation remains limited for several reasons. While biomaterial biocompatibility, immune regulation, and the utilization of stem cells have received considerable attention, the mechanical environment of the graft–host junction (GHJ), particularly the mechanical mismatch between grafts and host tissues, has not been paid enough attention. This review proposes that mechanical continuity is as fundamental as biological compatibility for durable graft integration and examines the GHJ as a spatiotemporally evolving mechanobiological system coupled with immune surveillance and responses. In this review, we first draw lessons from two natural tissue transitions that involve changes in mechanical properties: the development of the muscle–tendon–bone unit and skin-wound healing processes. We then survey current clinical applications of soft-tissue TECs. It comes out that complications frequently arise at or near the graft–host junction; however, the limited clinical characterization of GHJ mechanics also indicates that interface mechanobiology remains substantially overlooked in TEC clinical applications. In view of this situation, we analyze the potentially critical roles of mechanobiology at the GHJ, characterizing graft integration as intertwined spatiotemporal processes governed by both general and mechano-immune coupling. We finally advocate for incorporating mechanobiological considerations into graft interface design to achieve functional integration in clinical TEC applications and highlight AI-assisted inverse design and multimaterial 3D bioprinting as the most promising technologies to realize mechanobiological design and fabrication of the TEC junction portion.

## 1. Introduction

During the past three decades, remarkable advances in developmental biology, stem-cell biology, biomaterials, and biofabrication have enabled tissue engineering to create increasingly sophisticated biological substitutes for damaged tissues and organs [1,2,3,4,5]. Tissue-engineered constructs (TECs) now encompass a broad spectrum of products entering preclinical or clinical application [6,7,8,9,10,11]. Unlike conventional prosthetic implants [12,13,14,15] that primarily restore structural integrity, TECs are expected to participate actively in biological remodeling after implantation. Their long-term success therefore depends not only on the initial properties achieved during in vitro fabrication but also on their ability to interact dynamically with the host microenvironment. Following implantation, TECs are immediately exposed to inflammatory responses, immune surveillance, biochemical signaling, and continuous mechanical loading. Consequently, implanted grafts undergo a prolonged remodeling process during which both the graft and the surrounding host tissue continuously evolve [3,16,17]. Despite remarkable advances in this multidisciplinary field, the long-term clinical performance of many TECs remains considerably below expectations. Clinical complications including chronic inflammation, thrombosis, immune rejection, mechanical failure, and progressive functional deterioration continue to limit widespread clinical application [16,17,18,19,20,21,22].

This discrepancy highlights that the principal challenge is no longer solely the construction of functional tissues in vitro, but rather the establishment of stable integration between TECs and their physiological environment in vivo. Regardless of tissue type or implantation site, every engineered construct must eventually become incorporated into the host through continuous biological adaptation, in which the graft–host junction (GHJ) plays a critical role [17,23]. GHJ represents the region where engineered and native tissues first establish physical and biological communication. Traditionally, this interface has often been regarded as a simple geometric boundary separating two materials. In reality, however, it constitutes a highly dynamic transitional zone in which multiple species of cells, ECM components, newly formed blood vessels, and remodeling scaffold materials coexist and continuously interact. Rather than remaining a static boundary, the anastomotic region gradually evolves into newly organized tissue whose composition, architecture, and mechanical properties differ from both the original graft and the native host tissue. Clinical observations consistently demonstrate that complications frequently localize near GHJs [17,24,25,26,27], which suggests that this graft–host transitional zone represents one of the principal determinants for implantation success.

These complications are closely related to several forms of discontinuity coexisting within the transitional zone. Structural discontinuity results from interruption of native tissue architecture. Cellular discontinuity reflects differences in resident cell populations between graft and host. Biochemical discontinuity arises from differences in ECM composition, cytokines, and growth-factor distributions. Vascular discontinuity limits oxygen and nutrient transport before neovascularization is established. In electrically active tissues, electrophysiological discontinuity may further impede signal propagation [28]. Superimposed upon all these factors is mechanical discontinuity, which governs how physiological loads are transferred across the GHJ. Mechanical differences are inevitable because synthetic and naturally derived biomaterials often possess intrinsic properties that differ substantially from those of native tissues [29,30,31,32,33,34]. Consequently, physiological loading is redistributed across the GHJ, generating nonuniform stress and strain fields. Figure 1 shows a simulation example on the stress concentration at a vascular graft–host anastomotic junction as the graft vessel is 10 times stiffer than the native host, which results in pronounced stress concentrations at the anastomosis under axial extension or bending. These localized stresses are repeatedly imposed on cells and ECM near the junction, creating a mechanical microenvironment that differs markedly from that in either the graft or the native vessel. Stress concentration is also evident near suture lines [35,36], even for the implantation of decellularized native constructs that have similar mechanical properties to their native counterparts.

Therefore, abrupt changes in mechanical properties reshape the local mechanical microenvironment experienced by resident cells, and thereby regulating mechanotransduction, inflammatory responses, ECM remodeling, angiogenesis, and ultimately functional tissue regeneration [29,37,38,39,40]. Understanding how these events are coupled at GHJs provides a new conceptual framework for interpreting graft–host integration and may establish new design principles for next-generation regenerative products.

Historically, stress concentrations have primarily been considered as engineering problems associated with structural durability or mechanical failure. In recent years, Mechanobiology has emerged as one of the central paradigms of modern cell biology. Virtually every cell type involved in tissue regeneration—including fibroblasts, endothelial cells, vascular smooth muscle cells, macrophages, and mesenchymal stem cells—possesses specialized molecular machinery capable of detecting changes in its mechanical microenvironment [41,42,43,44,45,46]. Receptors such as integrins and ion channels Piezo1 and TRPV4 transmit extracellular mechanical cues to intracellular signaling pathways and ultimately to the nucleus, where gene expression is remodeled and consequently cell proliferation, migration, differentiation, and ECM synthesis are regulated [40,41,42,43]. Accumulating evidence indicates that mechanical signals also regulate immune responses during tissue repair. Macrophages exhibit remarkable mechanosensitivity such that substrate stiffness, matrix architecture, and mechanical loading influence macrophage polarization toward pro-inflammatory (M1-like) or pro-regenerative (M2-like) phenotypes [44,45]. Consequently, changes in the mechanical microenvironment can profoundly influence tissue regeneration without altering the underlying biochemical composition.

Despite these impressive developments in mechanobiology, the initiating role of mechanical discontinuity at GHJ in graft–host integration has received comparatively little clinical attention. In this review, we adopt a mechanobiological perspective to examine the graft–host transitional zone as a dynamic biological system rather than a static structural boundary. We first summarize naturally occurring biological interfaces that achieve seamless mechanical integration during development and tissue repair. We then survey the current TECs in clinical application to analyze the potentially critical roles of mechanobiology in the GHJ integration. We advocate incorporating mechanobiological considerations into graft interface design to achieve functional integration in clinical TEC applications. To this end, we characterize graft integration as an intertwined spatiotemporal process governed by both general and mechano-immune coupling. Accordingly, engineering strategies for functional interface design should emphasize spatially graded mechanics and temporally adaptive, remodeling-responsive matrices. We particularly highlight AI-assisted inverse design and multimaterial 3D bioprinting as the most promising technologies to realize mechanobiological design for TEC junction portions.

## 2. Lessons from Nature: Two Examples of Native Tissue Junctions

In this section, we examine two examples of naturally occurring junctions, respectively, in natural development and tissue repair. We reveal how graded structures, cellular adaptation, matrix remodeling, and immune regulation accommodate differences in tissue mechanical properties. These natural models therefore provide benchmarks for understanding failures and improvements required in clinical TEC implantations.

### 2.1. Muscle–Tendon–Bone Unit

As shown in Figure 2, the tendon forms a mechanically graded linkage that transfers contractile force from compliant muscle to much stiffer bone while minimizing stress concentration. Instead of an abrupt connection, the muscle–tendon–bone unit exhibits progressive transitions in interfacial geometry, collagen organization, cell phenotype, matrix composition, mineralization, and stiffness.

At the myotendinous junction, elongated muscle fibers terminate in deeply folded, finger-like membranes that interdigitate with tendon collagen fibrils. These folds enlarge the contact area and therefore reduce local stress as the force passes through adhesion complexes into the collagen-rich matrix. Concurrently, the dominant cells transition from contractile myocytes to fibroblast-like tendon cells. Within tendon proper, type-I collagen fibrils form parallel fibers and fascicles aligned with the loading direction. Spindle-shaped tenocytes maintain this tensile matrix, while tendon stem/progenitor cells support remodeling and repair. Physiological loading promotes tenogenic differentiation, whereas abnormal loading can produce inappropriate phenotypes and disorganized matrices [24,47]. Near bone, the enthesis progresses through tendon, unmineralized fibrocartilage, mineralized fibrocartilage, and bone. Collagen becomes less uniformly aligned, elongated tenocytes transition into rounder fibrochondrocytes, and the matrix becomes enriched in type-II and type-III collagen and proteoglycans, accommodating tensile, compressive, and shear deformation [48,49]. Across the tidemark, increasing mineralization, altered collagen organization, and hypertrophic chondrocyte-like cells culminate in mineralized collagen interdigitating with bone. Ligament–bone interfaces exhibit comparable transitions in composition, mineral distribution, and mechanics as well [49,50]. This natural gradient transition allows stiffness to rise progressively from tendon to bone and reduces localized stress at their otherwise severe mechanical mismatch. Its importance is reinforced by tissue-engineering studies: multilayer collagen scaffolds supporting fibroblasts, chondrocytes, and osteoblasts in corresponding regions better reproduce the native tendon–bone transition than homogeneous scaffolds [51,52].

### 2.2. Wound Healing of Skin

Skin wound healing is a rapid, evolutionarily conserved response that restores barrier integrity and prevents infection. It comprises four overlapping phases—hemostasis, inflammation, proliferation, and remodeling—during which cellular composition, ECM, and mechanical properties continually change [53,54,55,56]. Unlike the gradual development of the muscle–tendon–bone junction, adult healing prioritizes rapid closure and survival, often producing a structurally and mechanically imperfect scar rather than true regeneration [55].

As illustrated in Figure 3, healing is a temporally coordinated, immune-activated repair process. Immediately after injury, vessels constrict and platelets form a fibrin-rich clot that arrests bleeding and provides a provisional matrix containing chemokines and growth factors [53,54,55,56]. Within hours, damage-associated signals, complement, and microbial products recruit neutrophils, which remove bacteria and damaged tissue through phagocytosis, oxidants, and proteases [53,54]. Monocytes subsequently differentiate into macrophages. Their functions progressively shift from inflammatory defense and debris clearance toward inflammation resolution, angiogenesis, fibroblast recruitment, and matrix deposition, representing a phenotypic continuum rather than a binary M1/M2 switch [54,57]. In resolving wounds, neutrophils decline and macrophages become increasingly reparative. Conversely, infection, ischemia, diabetes, foreign materials, or repeated mechanical injury can sustain inflammation. Persistent proteases and oxidants degrade growth factors and newly deposited ECM, trapping chronic wounds in a non-resolving state [53,57]. Thus, acute inflammation initiates healing, whereas chronic inflammation reflects failure to transition efficiently from defense to tissue reconstruction. These immune responses closely resemble and share many features with those occurring at the GHJ following implantation.

Throughout wound healing, accompanied by active and dynamic immune responses, is the development and accommodation of mechanical discontinuity. As illustrated in Figure 4, beginning two to several days after injury, keratinocytes migrate and proliferate to restore epidermal continuity. Endothelial cells and pericytes form microvessels, generating vascularized granulation tissue [58,59]. Meanwhile, fibroblasts enter the fibrin–fibronectin matrix and deposit fibronectin, proteoglycans, and predominantly type III collagen. Granulation tissue is initially hydrated, porous, compliant, and mechanically weak. However, fibroblasts generate traction through integrin–cytoskeleton connections, reorganizing and compacting the surrounding matrix [58,59,60]. Consequently, its local mechanics are highly heterogeneous: collagen near contracting fibroblasts becomes densified, aligned, and reinforced, whereas more distant regions remain compliant [60]. In response to transforming growth factor-β, matrix tension, and other signals, fibroblasts can differentiate into α-smooth-muscle-actin-expressing myofibroblasts. These cells contract the wound and compact the ECM, accelerating closure but potentially causing scar contracture when their activity persists [58,59,61]. A positive-feedback loop may develop: increasing matrix stiffness and external tension promote fibroblast activation, which enhances collagen deposition and contraction, further increasing local stiffness [58,59]. Over subsequent weeks to months, cellularity and vascularity decline, many myofibroblasts undergo apoptosis, and type III collagen is progressively replaced by thicker, cross-linked, and directionally aligned type I collagen [53,54,55,56], all of which are postulated as partially induced by the stiffness increase. Although stiffness increases, the scar does not recover the basket-weave collagen architecture, appendages, vascular organization, and complete mechanical function of normal skin. Scar strength remains lower than that of normal skin, while its stiffness may be higher; therefore, strength and stiffness are not synonymous in this situation.

Mature scars exhibit altered viscoelasticity, anisotropy, extensibility, and stress relaxation. Their abrupt connection to compliant normal dermis produces non-uniform strain and stress concentrations during movement. Comparable mechanical effects of fibrotic scarring occur in other active tissues, including myocardium [62]. Excessive loading can sustain mechanotransduction, fibroblast activation, and inflammation. The proposed “stiffness-gap” mechanism links mismatch between ECM stiffness and cellular mechanics to keloid progression [63]. Moreover, inhibiting mechanotransduction in split-thickness skin grafting reduces fibrosis and contracture, demonstrating that mechanical signaling is a modifiable driver of scarring [61]. Thus, rapid healing closes a defect without necessarily eliminating mechanical mismatch. Similarly, differences in modulus, anisotropy, degradation, and stress concentration may determine whether the GHJ zone undergoes integration or fibrosis.

Cellular senescence has a phase- and context-dependent role in wound healing. Transient senescent fibroblasts and endothelial cells appear after injury and release senescence-associated secretory phenotype (SASP) factors, which promote myofibroblast differentiation and wound closure. Later, myofibroblast senescence can restrict extracellular matrix deposition and fibrosis [64,65]. Conversely, persistent senescent fibroblasts and macrophages in aged or diabetic wounds sustain inflammatory SASP signaling and are associated with delayed pathological repair [66]. Mechanical wounding itself induces rapid senescence, while 15% cyclic stretch at 15 cycles/min for 24 h induces epithelial senescence in lung models; secretions from these cells subsequently activate fibroblasts [67,68]. These findings support extending this framework to engineered skin, vessels, bladder, airway, cardiac and tendon tissues. Scaffold stiffness, strain, pressure and shear may regulate senescence and SASP activity at the graft–host junction, thereby influencing inflammation, fibrosis, vascularization and functional integration.

## 3. Current Situation of Clinical Applications of Soft-Tissue TECs and the Clues of Mechanobiological Importance

### 3.1. Skin Substitutes

Skin substitutes were among the earliest TECs to reach clinical application. Burke and colleagues reported a collagen–glycosaminoglycan dermal template covered by a silicone layer for extensive burn wounds in the early 1980s [69]. Cellular constructs followed, including Dermagraft, comprising allogeneic fibroblasts on a biodegradable mesh, and the bilayered fibroblast–keratinocyte construct Apligraf. Randomized studies demonstrated that these products could improve healing of venous leg ulcers and diabetic foot ulcers when added to appropriate standard care [70,71]. Skin substitutes now include cellular or acellular, autologous, allogeneic, xenogeneic, and synthetic products and are used principally for burns and chronic diabetic, venous, and surgical wounds [72]. Nevertheless, no available construct completely reproduces native skin. Important limitations include infection, variable graft take, contraction and recurrence, incomplete restoration of appendages, pigmentation and sensation, short shelf life of living products, manufacturing variability, and high cost [72,73,74,75,76].

Mechanobiological factors can reasonably be expected to influence the performance of tissue-engineered dermal substitutes in restoring skin structure and function. A small but informative body of preclinical and in vitro evidence indicates that graft–host integration is mechanobiologically regulated. By fabricating oligomeric collagen dermal scaffolds under controlled compression, Sohutskay et al. [75] demonstrated that increasing the fibril content of the scaffolds inhibited wound contraction and reduced the expression of myofibroblast markers. Cellular and vascular infiltration in a rat model also varied according to the graded density and orientation of the collagen fibrils. This research group subsequently developed a mechanobiological computational model to support the design and evaluation of next-generation regenerative dermal scaffolds fabricated from polymerizable collagen. The model captures the relationships between multiscale scaffold biomechanics and cell–scaffold mechanochemical interactions [76]. To overcome the substantial contraction of engineered skin substitutes, Pontiggia et al. [77] developed an anti-shrinkage device that imposed physical constraints on the constructs. This approach mitigated contraction and enhanced the stability and reproducibility of bioengineered skin substitutes for preclinical applications. These findings are consistent with established principles of wound mechanobiology: matrix stiffness, tension, compression, shear, and cyclic deformation regulate fibroblast migration, myofibroblast differentiation, collagen organization, angiogenesis, wound contraction, and scar formation. The mediators include integrins, focal adhesions, cytoskeletal tension, YAP/TAZ, and related pathways [58,59]. Excessive graft contraction can itself generate tension in adjacent host tissues, potentially causing functional impairment and contracture [73].

Nevertheless, the mechanobiology of the graft–host transitional zone remains an overlooked subject in clinical skin-substitute research. Most trials report complete closure, healing time, graft take, keratinized-tissue width, appearance, infection, and adverse events, but rarely measure spatial modulus gradients, interfacial strain transfer, residual tension and stress, or mechanically activated signaling. This omission probably reflects the historical priority of achieving rapid wound coverage and preventing infection, the difficulty of performing repeated mechanical measurements in fragile wounds, and the lack of standardized clinical mechanobiological endpoints.

### 3.2. Tissue-Engineered Vascular Grafts (TEVGs)

Vascular graft engineering began with Weinberg and Bell’s three-layer vascular model in 1986 [78]. The first clinical implantation of a tissue-engineered pulmonary artery was reported by Shin’oka and colleagues in 2001 [79], followed by biodegradable, bone-marrow-cell-seeded conduits and patches for congenital cardiovascular reconstruction [80]. At a later follow-up of 25 extracardiac cavopulmonary conduits, no graft-related mortality, rupture, infection, aneurysm, or ectopic calcification was observed; however, four patients developed stenosis requiring angioplasty, establishing stenosis as the principal graft-related failure mode [79].

Other strategies have included scaffold-free autologous vessels, biodegradable polymer scaffolds designed for in situ regeneration and decellularized human ECM conduits [81,82,83,84]. In December 2024, the US FDA approved SYMVESS, the first acellular TEVG, for adult extremity arterial injuries when urgent revascularization is required and an autologous vein is unavailable [85]. Nevertheless, clinical translation remains restricted to selected indications. Small-diameter arterial grafts always remain especially challenging because of issues such as thrombosis, insufficient endothelialization, aneurysmal dilation, calcification, and manufacturing complexity [86].

In clinical implantation of conventional vascular grafts and TEVGs, it seems that stenosis is not invariably an anastomotic phenomenon. In the Japanese pediatric cohort, Sugiura et al. explicitly reported that there was “no tendency” regarding stenosis location: stenosis occurred either at an anastomosis or within the mid-graft [87]. Meanwhile, clinical experience with conventional graft implantation in one of the authors’ groups suggests that, although anastomotic stenosis may occur slightly more frequently, it is insufficient to conclude that anastomotic stenosis is more likely to occur than graft-body stenosis [88,89]. Similarly, a large-animal study found stenosis localized to the mid-distal graft, whereas no change was detected at the proximal anastomosis [90]. Thus, current evidence does not justify a general statement that restenosis is more frequent at anastomoses than at the graft mid-portion. Instead, at least two partially distinct phenotypes should be recognized: diffuse or mid-graft narrowing arising during scaffold degradation and neotissue formation, and focal anastomotic overgrowth arising within the GHJ transitional zone. The second phenotype is illustrated particularly clearly in the discussion following the early clinical study by Shin’oka et al. [80]. The authors attributed recurrent pulmonary restenosis to tissue overgrowth at the anastomosis where suture material remained and admitted, importantly, “no solution for tissue overgrowth at the anastomosis site” [80], which reflects the reality of current clinical practice and a direct surgical recognition that the interface—not merely the graft material—is biologically active and clinically vulnerable.

Mechanobiologically, endothelial cells, smooth-muscle cells, fibroblasts, and macrophages near the anastomosis experience a combination of altered shear stress, cyclic strain, matrix stiffness, and inflammatory signaling that is not present in the graft mid-portion. These stimuli are mutually reinforcing. Altered wall strain and increased substrate stiffness favor smooth-muscle-cell migration, proliferation, and matrix synthesis; suture-associated macrophages release TGF-β and other profibrotic mediators. The resulting wall thickening further narrows the lumen and changes shear stress, establishing a feedback loop. Experimental findings showed that macrophage infiltration correlated with vascular graft stenosis, while reducing macrophage recruitment decreased stenosis but also impaired neovessel formation [91]. Computational and experimental studies further indicate that early neotissue formation is predominantly inflammation-driven, whereas later remodeling increasingly becomes mechano-mediated as the scaffold degrades and resident cells carry more hemodynamic load [90,92]. Patient-specific imaging demonstrated a compliance mismatch from a relatively stiff polymeric scaffold—approximately 4.3-fold stiffer than the native inferior vena cava—to a more distensible neovessel [93]. This mismatch is therefore biologically plausible and experimentally associated with anastomotic intimal hyperplasia [94], but it should not be presented as an isolated sufficient cause. The clinically relevant mechanism is more likely a multi-hit interaction among compliance and diameter mismatch, suture constraint, surgical injury, foreign-body inflammation, scaffold degradation, and patient-specific healing.

Surgeons have thus recognized anastomotic overgrowth, but current treatments—surveillance, balloon angioplasty, and occasionally stenting—mainly correct the resulting narrowing rather than its mechanobiological cause [80,81,87,93,95]. A genuine solution will require transitional-zone design: gradual compliance and reduced suture-associated rigidity, controlled local immunomodulation, optimized anastomotic geometry, and degradation synchronized with mechanically competent neotissue formation. The GHJ should therefore be designed as a spatially and temporally adaptive tissue region, rather than treated as a geometrically negligible surgical seam.

### 3.3. Urinary Bladder

Bladder tissue engineering developed from early attempts to replace the bladder wall with synthetic materials or acellular tissues. A major clinical milestone was the implantation of autologous urothelial- and smooth-muscle-cell-seeded collagen or collagen–polyglycolic acid scaffolds in seven children requiring augmentation cystoplasty [11]. The best improvements in capacity and compliance occurred with composite scaffolds wrapped in vascularized omentum. However, a subsequent phase II study found neither significant improvement in compliance nor capacity and reported an unacceptable frequency of serious adverse events [96]. Thus, bowel-based augmentation remains the clinical standard, and no tissue-engineered bladder substitute is routinely available [97].

A successful TEC bladder requires rapid urothelial barrier formation, organized smooth muscle, vascularization and innervation, fatigue resistance, native-like anisotropy and compliance, and degradation synchronized with neotissue maturation [97,98]. In the initial clinical series, cystography at three weeks showed no urinary leakage, and the graft–native margins later became cystoscopically indistinguishable [94]. These results demonstrate that satisfactory integration is possible, but they may be attributed to prolonged catheter drainage, fibrin sealing, and omental support, which mechanically and biologically protected the interface during early healing. Leakage and perforation are reported among potential complications of incomplete regeneration, ischemia, inadequate barrier formation, and scaffold failure [97,98], but interfacial stiffness, strain, or stress concentration has rarely been measured clinically. Therefore, a direct causal claim for mechanical mismatch induction would exceed the available evidence.

Nevertheless, the mechanical-mismatch mechanism is compelling. During filling, the native bladder expands markedly, whereas a newly implanted scaffold may be stiffer, less anisotropic, or less extensible. Sutures locally constrain expansion and concentrate tensile and shear stresses at the graft edge. Repeated filling can consequently generate micromotion, suture-hole enlargement, local ischemia, and epithelial discontinuity. Once urine enters the incompletely integrated wall, its cytotoxic and inflammatory effects may deteriorate cell survival and delay matrix re-organization. Surgeons have implicitly recognized this vulnerability. Catheter drainage, fibrin glue, omental coverage, and delayed bladder cycling are used to unload and vascularize the anastomosis [94]. Closely related evidence for mechanical mismatch in urethras reconstruction is particularly instructive: after catheter removal at two weeks, one engineered urethra developed narrowing at the proximal graft anastomosis; subsequent patients retained catheters for four weeks [99,100]. It shows clinical recognition that premature mechanical loading can compromise a tissue-engineered urinary interface.

### 3.4. Trachea and Airway

Short tracheal lesions can usually be treated by stent enhancement and resection with tension-free end-to-end anastomosis, whereas long-segment defects remain exceptionally difficult [101,102]. Conventional approaches include autologous reconstruction, stenting, and, in highly selected cases, vascularized tracheal allotransplantation [103,104]. No tissue-engineered circumferential tracheal replacement is currently established for routine clinical use [105].

In 2008, a decellularized donor trachea seeded with autologous epithelial cells and mesenchymal-stromal-cell-derived chondrocytes was reported as a successful “tissue-engineered airway” [106]. Synthetic stem-cell-seeded grafts were subsequently implanted and described as functional regeneration [107]. These reports attracted exceptional scientific and public attention. However, later investigations found discrepancies between published claims and clinical outcomes; the principal reports were ultimately retracted in 2023. Long-term analysis of three synthetic-graft recipients documented repeated interventions, anastomotic fistulae, obstructive granulation tissue, graft dehiscence, bleeding and air leakage, without vascularization, mucosal lining, or integration into surrounding tissue [108].

Large-animal studies reproduced the clinical failure pattern. Stenosis occurred at both proximal and distal anastomoses in every evaluated graft; the minimum lumen was located at the proximal anastomosis in 43% of animals [109]. Another study found stenosis most severe at both anastomoses, while neotissue formation remained limited to the graft margins and consisted predominantly of inflammatory granulation tissue rather than functional airway tissue [110]. Cell seeding was delayed but did not prevent stenosis [111]. These studies provide direct evidence that complications concentrate at the GHJs and explicitly identified supraphysiological scaffold properties and compliance mismatch as potential causes of stenosis or graft failure [110]. Future grafts require prevascularization, rapid mucociliary coverage, infection control, native-like anisotropic mechanics, fatigue resistance, and graded transitions in stiffness and geometry [112].

### 3.5. Other Emerging Clinical Applications of TECs and Translational Researches

Cardiac tissue engineering has progressed clinically furthest in recent years. Chachques and colleagues implanted a collagen matrix containing autologous bone-marrow cells over infarcted myocardium during coronary bypass surgery [113]. Increased scar thickness and improved regional function were reported. Subsequent injectable matrices, cellular sheets, and epicardial patches have likewise sought to limit ventricular dilatation and provide regenerative cells [114]. More recently, the phase 1–2 study implanted induced-pluripotent-stem-cell-derived engineered heart muscle in patients with advanced heart failure, providing clinical and histological evidence of graft vascularization and myocardial performance improvement [115]. Mechanobiologically, the cardiac graft–host interface is exceptionally demanding. It must transmit cyclic force while allowing electrical propagation and rapid vascular integration. A patch that is much stiffer than infarcted myocardium may reduce wall stress initially but concentrate strain at its margins; an excessively compliant or weakly attached construct may stretch, delaminate, or transmit little contractile force. Incomplete electrical coupling can additionally create conduction discontinuities and potentially arrhythmogenic boundaries. Thus, increased wall thickness should not alone be interpreted as functional integration: future trials should quantify interface strain, force transmission, conduction velocity, vascular perfusion, and their evolution during scaffold degradation [113,114,115].

In neural repair, commercially available nerve conduits and processed allografts are used for selected short peripheral-nerve gaps, whereas living, cell-seeded tissue-engineered nerve grafts remain predominantly experimental [116,117]. Neural cells respond strongly to substrate stiffness, topography, tension, and adhesion; these cues regulate growth-cone migration, neurite branching, and differentiation [116]. Consequently, abrupt differences in stiffness, diameter, fascicular organization, or strain between graft and nerve may disturb axonal trajectories and promote fibrosis. Excessively stiff conduits can also mechanically irritate adjacent tissue, whereas overly compliant conduits may collapse. For central-neural implants, mechanical mismatch with very soft neural tissue may perpetuate micromotion, glial activation, and encapsulation. Nevertheless, clinical nerve-repair studies rarely measure local interface mechanics, making mechanobiological graft integration an important but clinically underexamined endpoint [116,117].

Tendon and ligament reconstruction presents an explicit junction problem. Clinical matrices and patches are generally used as biological or mechanical augmentation; engineered, cell-containing, multilayer constructs that reproduce the enthesis remain mostly preclinical. Native tendon-to-bone attachment changes gradually from tendon to bone, thereby distributing load without a sharp stress concentration as explained in the above section [47,52,117,118]. In contrast, conventional graft fixation instead creates an abrupt material transition that commonly heals through mechanically inferior scar tissue [119,120]. Multilayer collagen scaffolds and mineral or structural gradients therefore attempt to reproduce this spatial transition and direct region-specific cell differentiation [52].

Most recently, Durkin et al. combined a size-matched decellularized scaffold, autologous cells, bioreactor conditioning, and temporary stenting to implant an autologous engineered esophagus in a large-animal model. This significant study showed that graft stiffness, strength, and extensibility progressively approached native esophageal values, vascularized neuromuscular tissue formed, and peristalsis was restored without anastomotic leakage or symptomatic stricture at six months [121]. Similarly, computationally compliance-matched vascular grafts maintained native-like distensibility and promoted contractile smooth-muscle remodeling at the anastomosis [122], while biodegradable grafts matured into growing, flow-responsive neovessels [123]. Collectively, these studies support mechanical compatibility and structural support as determinants of junctional continuity and host-driven functional regeneration.

### 3.6. Sectional Remarks

These clinical applications of TECs discussed above are summarized in Table 1. Clinical experience of soft-tissue TECs reveals a recurring discrepancy: the GHJ is widely recognized as a clinically vulnerable site. Anastomotic stenosis in TEVGs, leakage in urinary reconstruction, airway fistulae and obstructive granulation, skin-graft contraction, and tendon-to-bone failure all localize, or are particularly consequential, at mechanically discontinuous junctions. Such spatial correspondence constitutes important clinical evidence that interface mechanics matter. Surgeons clearly appreciate practical mechanical factors—suture retention, tension-free anastomosis, graft compliance, fixation stability, luminal continuity, resistance to collapse, and postoperative loading. Nevertheless, these factors are usually treated as surgical or material-design issues rather than as interconnected mechanobiological regulators of inflammation, cell migration, vascularization, fibrosis, and tissue remodeling. Thus, mechanobiology at GHJs remains an overlooked translational dimension. Practically, clinical studies prioritize survival, patency, leakage, luminal diameter, functional capacity, and adverse events; interface mechanics are difficult to measure in vivo. Meanwhile, failures are multifactorial, and trials frequently involve small heterogeneous cohorts without mechanically matched controls. Future TEC evaluation should combine clinical imaging with patient-specific computational analysis, elastography or deformation mapping, and spatial assessment of inflammation, fibrosis, vascularization, and matrix remodeling.

## 4. Mechanobiology of Graft–Host Integration: Intertwined Spatiotemporal Processes Coupled to Immune Responses

The GHJ is not a static boundary but an evolving transitional zone whose cellular composition, ECM, vascularization, immune state, and mechanical properties change continuously in space and time. Within this dynamic microenvironment, mechanical cues—including stiffness, strain, stress, topography, and interfacial micromotion—not only regulate immune-cell recruitment, activation, and phenotype but are themselves altered by inflammation, matrix dynamics, vascularization, graft degradation, and tissue remodeling. Mechanobiological factors therefore act simultaneously as regulators and outcomes of graft–host integration. Based on recent advances in mechanobiology, immunology, biomaterials, and regenerative medicine, this section discusses the potential roles of these reciprocal interactions in clinically relevant graft–host integration. Particular attention is given to distinguishing direct clinical evidence from experimental evidence and mechanistically supported inference. This discussion provides the conceptual foundation for translating mechanobiological knowledge into functional interface design strategies, as addressed in the following section.

### 4.1. Mechanoimmune Coupling at the GHJ

Implantation simultaneously produces tissue injury and introduces biological or synthetic material into the host. Immediately after implantation, blood-derived proteins adsorb onto the graft surface, and coagulation, complement activation, platelet adhesion, and release of damage-associated molecular patterns initiate acute inflammation [124]. Increased vascular permeability and endothelial activation facilitate leukocyte rolling, adhesion, and transmigration. Neutrophils generally arrive first, removing microorganisms and damaged material through proteases, reactive oxygen species, and extracellular traps; however, excessive or persistent neutrophil activity can damage the graft and surrounding viable tissue [125]. Monocytes subsequently differentiate into macrophages, which clear debris and coordinate inflammation, vascularization, ECM deposition, and remodeling. If the material cannot be phagocytosed, macrophages may fuse into foreign-body giant cells, while fibroblasts deposit collagen around the implant, potentially producing fibrous encapsulation [124,126,127]. Successful integration requires neither complete immune suppression nor unrestricted inflammation, but an appropriately timed transition from pathogen and debris clearance toward vascularization, matrix reconstruction, and functional remodeling.

The inflammatory sequence is not only governed by cytokines and graft chemistry, but also by mechanical factors. Leukocyte recruitment itself is mechanical-based: shear flow regulates selectin-mediated rolling and integrin-dependent arrest, while endothelial and basement-membrane mechanics affect transmigration [125,128]. Once within the interface, leukocytes must navigate a confined three-dimensional environment. T cells can undergo rapid amoeboid migration by deforming and squeezing through pre-existing collagen gaps, using fibrillar contact guidance without necessarily degrading the matrix [129,130]. Other leukocytes may become more adhesion-dependent in dense or injured tissues. Consequently, graft porosity and fiber organization regulate not only tissue ingrowth but also which immune cells can enter, how rapidly they move, and where they accumulate. Macrophages are particularly important mechanoimmune integrators. Integrin–cytoskeleton coupling, nuclear deformation, and mechanosensitive ion channels translate substrate mechanics into inflammatory or reparative programs. Constraining macrophage shape experimentally alters phenotype [131], while the mechanically activated channel PIEZO1 enables stiffness sensing and can promote inflammatory activation and fibrous-capsule formation [132]. Adaptive immunity is also force-sensitive. T-cell receptor recognition and integrin binding operate under cytoskeletal tension, and the duration of receptor–ligand bonds can depend on applied force [133]. Importantly, the mechanical anchorage of the ligand—not merely its biochemical density—affects signaling. Therefore, matrix remodeling or stiffening at the graft boundary could alter adaptive responses by changing the resistance against which immune receptors pull [134,135].

Mechanics can regulate immunity; meanwhile, immune reactions reconstruct the mechanical environment. For instance, neutrophil and macrophage proteases degrade ECM and scaffolds; macrophages and fibroblasts deposit collagen and generate contractile forces; and immune responses dominate scaffold degradation. These events progressively modify stiffness, porosity, and load transfer at the GHJ zone [126,136]. This reciprocal coupling produces a feedback loop, as illustrated by Figure 5.

Clinically, this loop offers a plausible common mechanism for the defects at GHJs described in the above section. For example, an abrupt stiffness mismatch may concentrate stress near sutures, causing repeated microinjury and maintaining inflammatory signaling; the resulting collagen deposition further stiffens the boundary and reinforces stress concentration. Conversely, an excessively compliant or rapidly degrading interface may permit micromotion, loss of sealing, and persistent macrophage activation. Nevertheless, direct clinical evidence linking measured interface mechanics to specific immune trajectories remains limited. Thus, mechanoimmune coupling is strongly supported experimentally and consistent with clinical complication patterns, but mechanical mismatch should currently be regarded as a plausible contributing mechanism rather than a universally demonstrated cause.

### 4.2. Spatial Mechanobiology: Mechanical Heterogeneity and Gradients Across the GHJ

Mechanobiology concerns how cells sense physical properties and convert them into biochemical signals that regulate migration, proliferation, differentiation, survival, and matrix production. Substrate flexibility regulates focal-adhesion formation and locomotion [137], such that fibroblasts encountering a stiffness boundary preferentially turn toward the stiffer region—a phenomenon subsequently termed durotaxis [138]. Individual focal adhesions can repeatedly “tug” on the ECM, with FAK–paxillin–vinculin signaling determining the stiffness range over which directional sensing occurs [139]. Mechanical information can subsequently reach the nucleus through cytoskeletal tension and regulators such as YAP/TAZ [41].

Spatial mechanics additionally influence cell phenotype. Matrix elasticity can bias mesenchymal-stem-cell differentiation toward neural-, muscle-, or bone-like lineages [38], whereas stress relaxation, ligand mobility, and porosity can substantially modify responses to nominally identical elastic moduli [140]. Therefore, “stiff” and “soft” are insufficient descriptions of an interface. Relevant parameters exhibited in natural materials include gradient magnitude, nonlinear and anisotropic behavior, viscoelastic relaxation, fiber alignment, local curvature, residual strain, and cyclic loading [141,142,143]. In 3D matrices, cells may switch between lamellipodial, lobopodial, and amoeboid migration according to adhesion, matrix architecture, RhoA–ROCK–myosin-II contractility, and proteolytic capacity [144]. T lymphocytes, for example, can migrate rapidly through collagen using transient, low-affinity interactions without classical focal adhesions [145]. Dendritic-cell migration varies with maturation and tissue origin, while aligned collagen directs migration by contact guidance [60,146]. Hence, interface architecture may spatially select not only stromal-cell ingrowth but also immune surveillance and antigen-presenting-cell trafficking. Fibroblasts embedded in graded collagen accumulated toward the stiffer region, demonstrating 3D durotaxis [147]. Importantly, recent in vivo experiments showed that stiffness gradients recruit fibroblasts toward fibrotic lesions, where FAK–paxillin and YAP signaling promote myofibroblast activation; disrupting this pathway reduced fibrosis [148]. Although this evidence derives from fibrosis and cancer rather than graft implantation, it provides a plausible mechanism by which a stiff implant margin or fibrotic capsule could continually attract matrix-producing cells.

At TEC implantation, the GHJ is commonly an abrupt mechanical boundary. In paired vascular grafts of animal implantation with similar luminal surfaces, compliant grafts showed substantially higher patency than stiff grafts, indicating that compliance mismatch can be a detrimental factor [149]. Computational and experimental analyses further suggest that mismatch alters suture-line stresses and may promote anastomotic intimal hyperplasia [150]. TEVGs also demonstrate that spatial biological and mechanical heterogeneity develop together. Neotissue formation depends on a balance between polymer degradation, immune-cell infiltration, host-cell recruitment, matrix deposition, and increasing transfer of hemodynamic load from scaffold to tissue. Modeling indicates that an excessive early inflammatory response can impair later mechanosensing and prevent the neovessel from approaching native mechanical properties [92]. Histological mapping also shows that the scaffold and luminal thrombus form distinct spatial compartments: early myeloid infiltration may predominate from the outer graft surface, whereas luminal thrombus can remodel into collagen-rich neotissue and contribute to obstruction [151]. In skin substitutes, differences in stiffness and prestress between graft, wound bed, and surrounding skin may spatially concentrate myofibroblasts and contraction at graft margins. Bladder and airway grafts combine compliance mismatch with sutures and repeated distension or bending, making the anastomosis susceptible to leakage, fistulae, granulation, and fibrosis. Neural tissues present an extreme mismatch: soft, stretchable neural interfaces produce less mechanical incompatibility than conventional rigid implants and improve long-term function in animal models [152].

### 4.3. Temporal Mechanobiology: Dynamic Remodeling and Mechanical Memory at the GHJ

The GHJ does not possess a static mechanical state but continuously evolves. Temporal mechanobiology therefore concerns not only which mechanical cues exist, but also their sequence, duration, rate of change, and relationship to cellular history. The behavior of mesenchymal stem cells (MSCs), which are regarded as promising cell components seeded in many clinical TECs, is specifically reactive to the mechanical dynamics of their environments. In hydrogels stiffened during culture, mesenchymal stem cells (MSCs) rapidly increased spreading and traction; early stiffening favored osteogenesis, whereas delayed stiffening favored adipogenesis [153]. Conversely, hydrogels designed to soften with time enhanced cardiomyocyte differentiation compared with persistently stiff matrices, indicating that developmental-like mechanical evolution can be more instructive than a constant modulus [154,155]. These findings are directly relevant to cells seeded within TECs and host progenitors recruited to their margins. Scaffold alignment and cyclic tensile stimulation can cooperatively guide MSCs toward a fibroblastic ligament-like phenotype even without additional differentiation factors [156]. Therefore, stem-cell fate at an interface may depend on whether mechanical loading begins before or after adhesion, vascularization, and matrix deposition. Furthermore, cellular responses may persist after the initiating mechanical environment has disappeared. MSCs maintained on stiff substrates retain nuclear YAP/TAZ activity and osteogenic commitment after transfer to softer matrices, with the duration of stiff “mechanical dosing” determining whether this response remains reversible [157]. MicroRNA-21 can maintain a fibrotic mechanical memory in MSCs after removal of the stiff stimulus [158]. Myofibroblasts previously cultured on stiff matrices likewise retain increased contractility when returned to softer environments [159], while epithelial cells primed on stiff substrates subsequently migrate faster on soft matrices through YAP-dependent memory [160]. At the GHJ, such memory implies that cells exposed initially to a rigid graft, high suture stress, or repeated micromotion may remain contractile or fibrogenic after the scaffold softens. Conversely, beneficial mechanical preconditioning of seeded stem cells may stabilize a regenerative phenotype after implantation.

Clinically, TECs’ mechanics evolve as the polymer loses load-bearing capacity and host-derived matrix assumes hemodynamic loading. Histological studies show that platelet-rich luminal material and the scaffold undergo distinct remodeling trajectories, with early myeloid infiltration followed by collagen deposition and smooth-muscle-like differentiation [151]. Most importantly, early vascular graft narrowing observed clinically and experimentally is not always a fixed failure. Serial imaging, animal validation, and computational modeling showed that inflammation-associated stenosis could subsequently regress as inflammation declined, collagen reorganized, and graft mechanics matured [90]. This does not imply that clinically significant stenosis should remain untreated; rather, it demonstrates that interface state and trajectory must be distinguished. A single measurement of luminal diameter, stiffness, or inflammation may not predict the final outcome [95,161,162]. Clinically successful integration should thus be defined not by immediate mechanical matching alone, but by coordinated evolution toward stable load sharing without persistent pathological mechanical memory.

## 5. Future Directions: Engineering Strategies for Functional Interface Design

The preceding clinical evidence suggests that a functional interface must regulate how forces, cells, immune signals, and matrix remodeling are transmitted across space and time [163]. For some of the clinical TECs discussed in Section 3, studies emphasizing mechanobiological considerations, particularly through the use of bioreactors, have demonstrated functional improvements, as summarized in Table 2. However, these studies remain limited to in vitro conditions and do not fully recapitulate the coupled immune and mechanical environment at the graft–host junction (GHJ). Future strategies should integrate three mutually dependent components: spatially graded mechanics, temporally adaptive behavior, and manufacturing methods capable of converting patient- and site-specific design into reproducible constructs.

### 5.1. Mechanical Gradient Engineering: Spatial Considerations

A sharp graft–host transition concentrates stress and strain, perturbs cellular mechanosensing, and can redirect immune-cell and stromal-cell migration. Mechanical-gradient engineering aims to distribute these changes over a finite transitional zone. Importantly, the design target should not be limited to a gradient in Young’s modulus. Native interfaces combine gradients in composition, viscoelasticity, anisotropy, porosity, fiber orientation, permeability, degradation rate, and cell-adhesive ligand density [163]. These parameters collectively determine local mechanical properties.

Continuously graded electrospun meshes have demonstrated the feasibility of spatially varying material composition across ligament–bone models [174]. Mineral gradients on nanofibrous scaffolds can likewise induce spatially graded osteogenesis rather than forcing an abrupt transition between unmineralized and mineralized phenotypes [175]. These findings support a broader graft–host principle: the spatial derivative of a mechanical property may be as biologically important as its absolute value. The optimal gradient will be tissue- and loading-specific. Vascular interfaces require circumferential and axial compliance gradients that preserve pulsatile haemodynamics; tendon or ligament interfaces require progressive mineralization and fiber reorientation; and cardiac, neural, bladder, and tracheal constructs require gradients in anisotropy, relaxation, and cyclic deformation. Gradient design should therefore be informed by multiaxial loading rather than a single uniaxial modulus. It must also accommodate biological remodeling: the initial gradient should not merely imitate mature tissue, but should anticipate scaffold degradation, host matrix deposition, and the resulting movement of the mechanical transition. Adaptive-scaffold concepts further suggest integrating spatial architecture with immunomodulatory and angiogenic functions [176,177].

### 5.2. Dynamic Interfaces: Temporal Considerations

A static “mechanically matched” implant may become mismatched within weeks because inflammation, degradation, fibrosis, vascularization, and tissue maturation continuously change interface mechanics. Dynamic interfaces should therefore be programmed around a desired mechanical trajectory: initial surgical strength and sealing, followed by controlled relaxation or degradation, cellular infiltration, matrix deposition, and eventual transfer of load to newly formed tissue.

Photodegradable hydrogels established that matrix mechanics and chemistry can be altered in the presence of cells with spatial and temporal control [178]. Cell-compatible stiffening systems subsequently showed that the timing of stiffening changes mesenchymal-stem-cell differentiation [153]. Time-dependent properties such as stress relaxation and creep are equally important. Rapidly relaxing hydrogels enhance cell spreading, proliferation, matrix production, and osteogenic differentiation independently of initial elastic modulus [140]. Furthermore, stem cells retain a mechanical memory of previous environments through YAP/TAZ-associated mechanisms, meaning that even a transiently inappropriate stiffness can influence later cell fate [157]. Interface design must therefore control not only the final modulus but also the magnitude, duration, sequence, and rate of mechanical cues.

### 5.3. Promising Manufacturing Technologies for Functional Interfaces: AI-Assisted Inverse Design and 3D Bioprinting

The design space of functional interfaces is too large for conventional trial-and-error optimization. Material composition and architecture, gradient length, degradation kinetics, and cell distribution interact nonlinearly and may produce conflicting outcomes. AI-assisted inverse design provides a promising technology for this complicated task. It should begin with desired performance—such as reduced interfacial stress, physiological compliance, controlled macrophage response, and tissue-specific differentiation—and infer combinations of material and architectural variables likely to achieve it. Machine-learning models have already predicted extrusion-print quality from biomaterial formulations and processing conditions, reducing experimental screening [179]. More advanced generative and active-learning frameworks can perform constrained multi-objective optimization of heterogeneous architectures, balancing modulus and manufacturability [180]. For GHJs, such models may integrate finite-element mechanics, degradation and tissue-growth models, and biological data describing inflammation, fibrosis, vascularization, and cell fate. Patient imaging and organ-specific loading could then define boundary conditions for individualized designs.

However, inverse design is only reliable within the domain represented by its training data. Small, heterogeneous animal datasets and poorly standardized mechanical measurements may generate apparently precise but biologically unreliable recommendations. Explainable models, uncertainty quantification, prospective validation, and iterative design–manufacture–test–learn cycles will therefore be essential [181].

Moreover, in biofabrication and mechanobiology, AI models may suffer from data scarcity due to the high cost and low throughput of reproducible human/animal dynamic mechanical testing. To overcome this training data bottleneck, one practical strategy can leverage physics-informed neural networks (PINNs) that incorporate mechanical conservation laws to reduce data reliance. First, PINNs and physics-informed graph networks can embed constitutive relations, incompressibility, compatibility, and boundary conditions into the loss function, excluding mechanically impossible predictions and reducing dependence on labeled data [182,183]. Second, multiplexed organ-on-chip arrays should systematically simulate graft–host environments. Automated stimulation, integrated sensors, live imaging, and standardized molecular endpoints can generate reproducible mechanobiological dose–response datasets across donors [184]. Third, calibrated finite-element, growth–remodeling, and agent-based models can generate synthetic parameter sweeps covering unsafe conditions. These data should pretrain AI surrogates and then explicitly estimate the simulation-to-reality discrepancy [185]. Finally, uncertainty-aware active learning should select the most informative experiments, creating a closed loop of simulation, chip validation, sparse animal confirmation, and model updating.

3D bioprinting provides the physical means to realize these predictions by positioning multiple biomaterials, cells, fibers, and vascular channels within one continuous construct. Embedded printing such as FRESH permits complex soft hydrogel structures that would otherwise collapse under their own weight during conventional extrusion [186], while stereolithographic methods have generated interconnected vascular and intravascular topologies in biocompatible hydrogels [187]. More recently, shear-extensional in situ particle-to-fiber transformation (SHIFT) has produced aligned, cell-scale hydrogel microfibers within printed filaments, promoting myotube alignment and volumetric muscle regeneration [188]. Such subvoxel anisotropy is particularly relevant because clinically functional interfaces require control from the organ scale down to cell-scale contact guidance.

As potential technologies for the engineering at GHJ, a dual-syringe extrusion printhead with a static or microfluidic mixer can continuously vary the host-like: graft-like bioink ratio along each toolpath, creating a transition zone rather than an abrupt material boundary. Kuzucu et al. demonstrated this approach using carboxylated-agarose inks, producing continuous 3D stiffness gradients of approximately 57–258 kPa alongside independently graded cell densities and immobilized peptides [189]. Mechanical anisotropy can additionally be graded by progressively changing filament orientation, spacing, and reinforcing-polymer content. Chae et al. combined a PU/PCL framework with tendon-derived, hybrid tendon/bone-derived, and bone-derived extracellular-matrix bioinks, achieving zone-specific differentiation, tendon–bone healing, and functional recovery in vivo [190]. For mechano-immune matching, an additional inlet could deposit a host-facing gradient of immunoregulatory cargo and stromal/vascular cells. As supporting evidence, BMP-4-loaded mesoporous silica nanoparticles incorporated into multicellular bioprints promoted M2 macrophage polarization, angiogenesis, and tissue regeneration [191]. Collectively, these studies support co-printing mechanical, cellular, and immunoregulatory gradients at the GHJ, although their combined application remains to be validated directly.

One of the ultimate strategies is therefore AI design plus advanced 3D bioprinting with the aid of clinical imaging and mechanical measurements. The technical roadmap is like individual measurements defining the problem; inverse models proposing a graded, temporally evolving architecture; multimaterial bioprinting fabricating a prototype; and biological/mechanical testing returning data to refine the model. This closed-loop framework could transform the GHJ from a surgically created discontinuity into a programmable transitional tissue.

## 6. Conclusions and Outlook

Successful soft-tissue implantation depends not only on the biological compatibility of the graft but also on the establishment and maintenance of mechanical continuity across the GHJ. The GHJ is not a fixed boundary created at implantation. It constitutes a dynamic and spatially heterogeneous mechanobiological system in which mechanical loading, immune responses, cellular migration and differentiation, scaffold degradation, vascularization, and ECM deposition continuously interact. Local mechanical properties can regulate the recruitment and phenotype of immune and reparative cells. Conversely, immune responses, matrix degradation, and tissue overgrowth progressively alter the mechanical conditions at GHJs. This bidirectional coupling can promote constructive remodeling, but it may also initiate self-reinforcing pathological loops, which in turn increase the discontinuities at the GHJs. Conventional evaluations of TECs have focused predominantly on cytocompatibility, immunogenicity, and the restoration of bulk-tissue function. Clinical experience, however, indicates that an implant may be biologically acceptable yet still fail because of compliance mismatch, stress concentration, micromotion, stenosis and leakage at GHJs. Future soft-tissue implants should therefore sufficiently take the GHJ design into account rather than single uniform replacement grafts. Such TECs should, by means of innovative technologies, incorporate graded mechanical properties to reduce abrupt discontinuities; introduce anisotropic architectures to reproduce direction-dependent force transmission; and enable dynamic remodeling capabilities that evolve with healing.

A considerable roadmap to realize this objective may be suggested as follows: (1) patient-specific imaging, multiaxial mechanical characterization, computational modeling, mechanobiological experiments, immune-competent tissue models, and longitudinal in vivo measurements are combined to define quantitative design targets; (2) AI-assisted inverse design identifies material–architecture combinations that satisfy competing mechanical and biological requirements; (3) multimaterial and cell-laden 3D bioprinting translate these solutions into spatially organized constructs. Undoubtedly, these technologies must nevertheless pass uncertainty assessment, clinically relevant testing, and manufacturing feasibility. Ultimately, the success of tissue-engineered implantation should establish a mechanically competent and biologically integrated functional tissue. Shifting the focus from the graft itself to the evolving GHJ zones offers a unifying framework for understanding current clinical complications and for developing the next generation of regenerative soft-tissue implants.

## Figures and Tables

**Figure 1:**
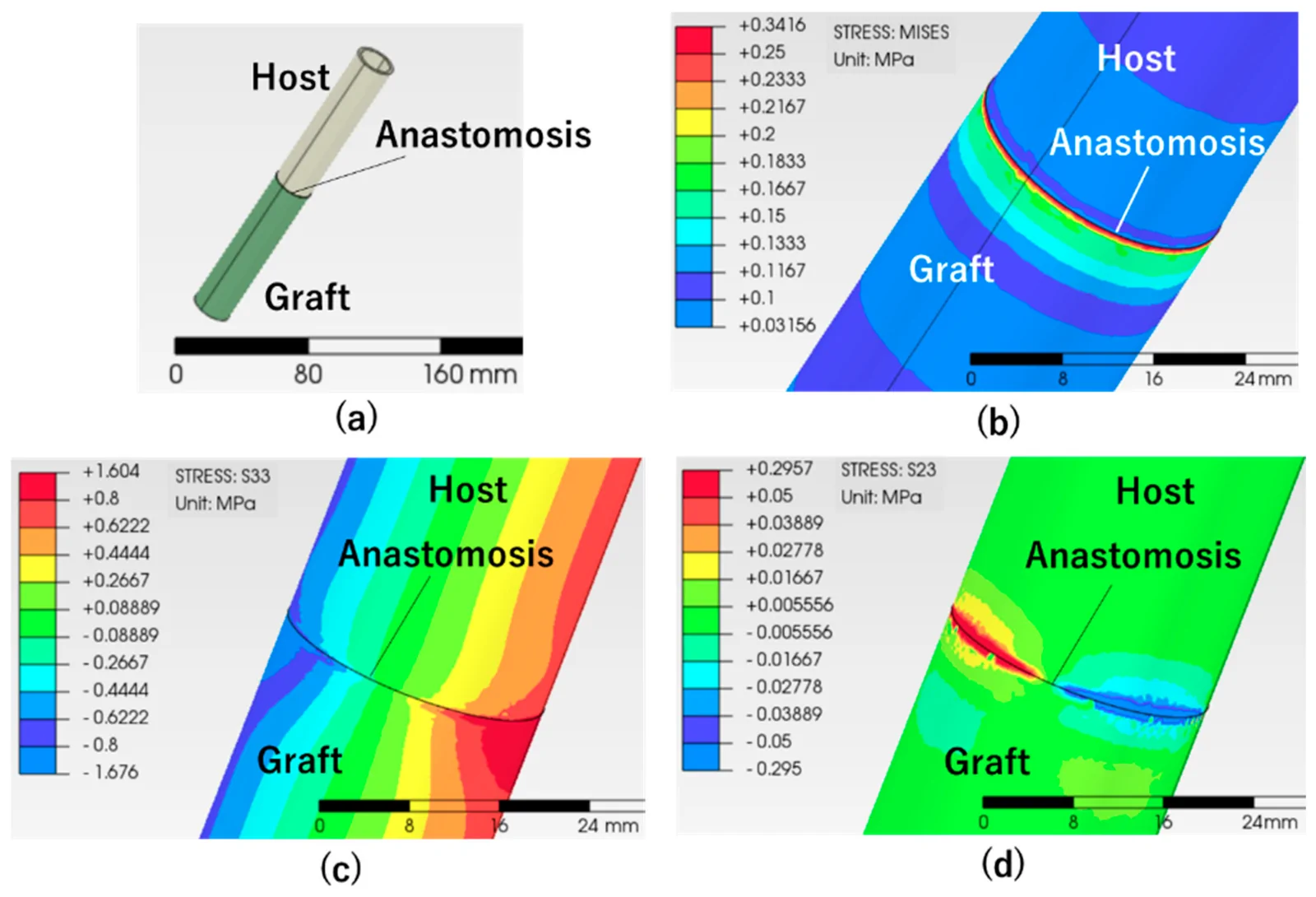
Computational simulation illustrating stress concentration induced by mechanical mismatch at the host–graft anastomosis following vascular graft implantation. (**a**) Schematic of the finite element model consisting of host and graft vessels with identical inner and outer diameters of 20 mm and 24 mm, respectively. Both the host and graft vessels have a length of 100 mm. The Young’s modulus is 0.5 MPa for the host vessel and 5.0 MPa for the graft vessel, while the Poisson’s ratio is 0.45 for both materials. (**b**) Distribution of von Mises stress at the anastomotic region when the host–graft construct is subjected to 10% axial elongation. (**c**) Axial normal stress and (**d**) circumferential shear stress distributions at the anastomosis under an applied bending moment of 500 N·mm at both ends of the structure. Simulations were performed using PrePoMax v2.5.0.

**Figure 2:**
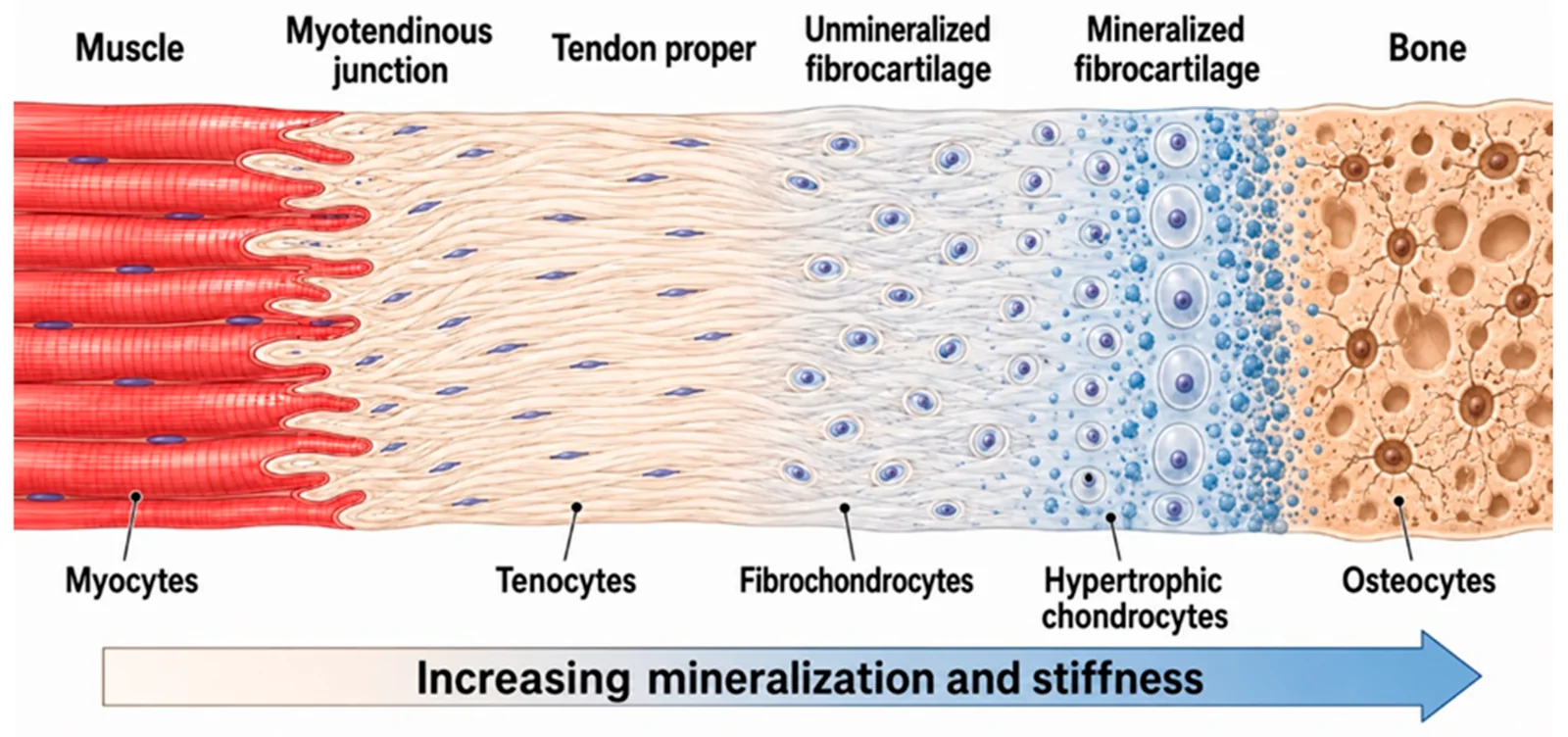
Schematic spatial transition from contractile muscle through the myotendinous junction and aligned tendon proper to unmineralized fibrocartilage, mineralized fibrocartilage, and bone. Cell morphology and phenotype change concurrently with collagen organization, mineralization, and stiffness.

**Figure 3:**
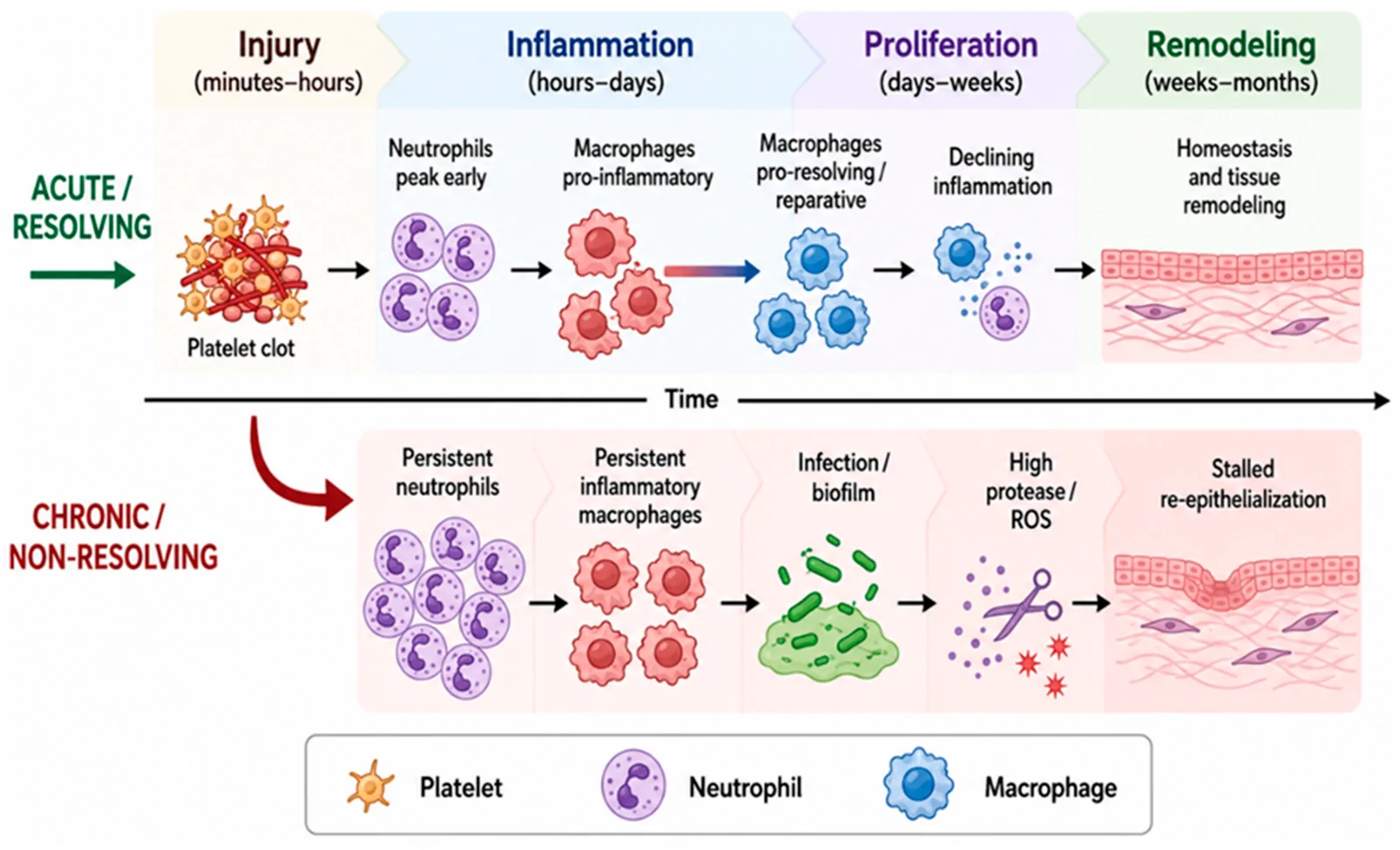
Temporal immune program of skin wound healing. The phases overlap substantially. Successful acute healing requires timely attenuation of neutrophilic inflammation and acquisition of pro-resolving and reparative macrophage functions. Persistent inflammatory cells, infection, proteases, and reactive oxygen species can produce a chronic, non-healing trajectory.

**Figure 4:**
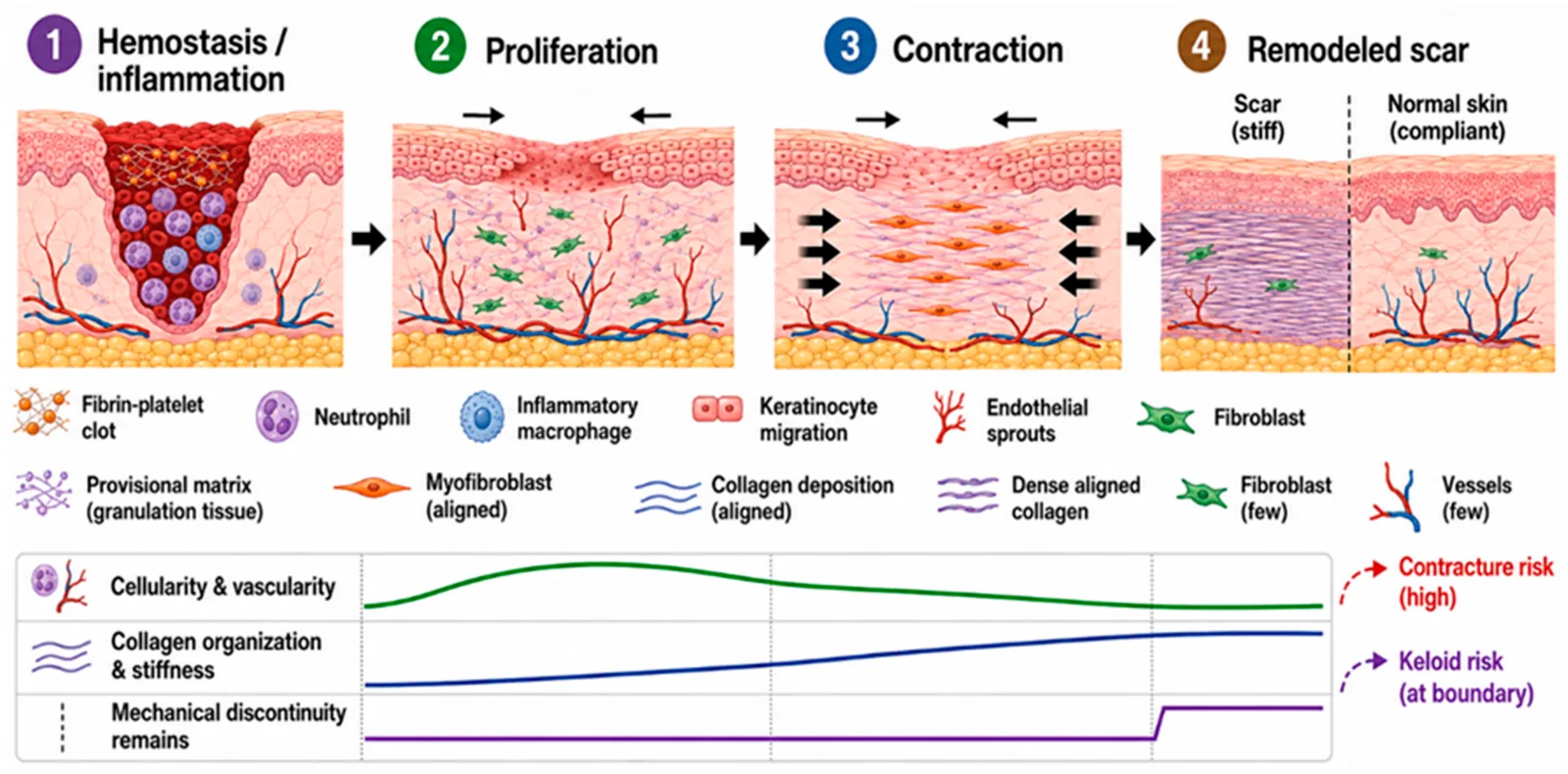
Cellular and mechanical evolution of a skin wound. The fibrin clot is replaced by vascular, cell-rich, compliant granulation tissue; fibroblast and myofibroblast traction then compacts and aligns collagen. Remodeling reduces cellularity and vascularity but increases collagen organization and local stiffness, leaving a mechanically discontinuous scar–normal-skin boundary.

**Figure 5:**
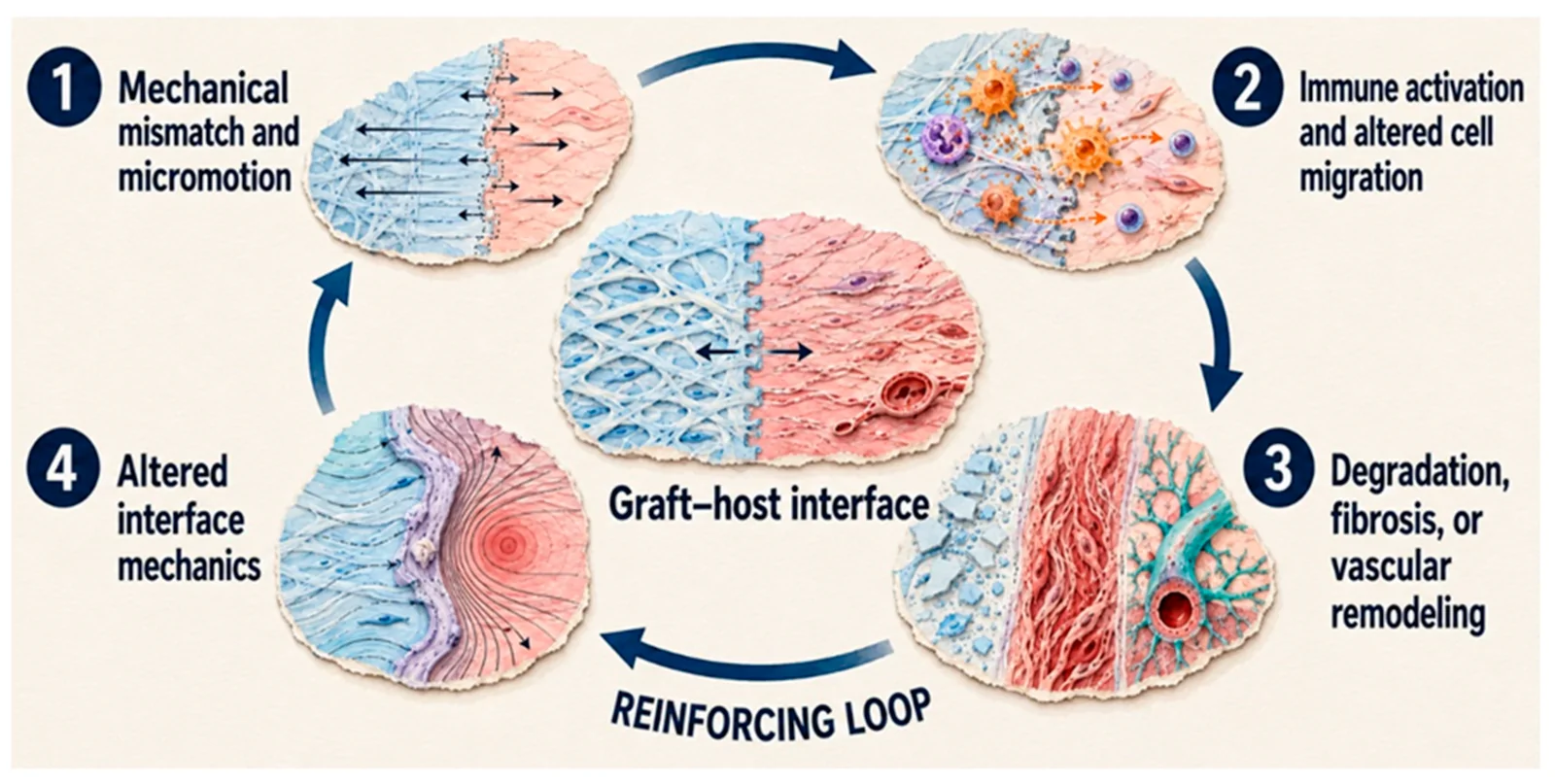
Mechanoimmune coupling generates a reinforcing feedback loop at the GHJ. Mechanical mismatch creates local stress/strain concentrations and matrix deformation, which activate immune cells and alter their migration across the interface. The resulting inflammatory response can drive scaffold degradation and tissue remodeling. These processes modify the stiffness, architecture, and force transmission of the interfacial tissue, thereby further altering local mechanics and perpetuating the mechanoimmune feedback loop. The illustrated pathways are interconnected and may occur concurrently rather than as a strictly sequential process.

**Table 1:** Clinical applications of TECs and the potential for graft–host junction mechanobiology.

Clinical Application	Principal Achievements	Remaining Challenges	Clues/Implications of Mechanobiology at the Graft–Host Junction	Refs
Skin substitutes	Commercial cellular and acellular substitutes improve wound coverage, granulation, and healing of burns and chronic wounds.	Delayed vascularization, infection, incomplete appendage regeneration, contraction, scarring, variable durability, and imperfect attachment to the wound bed.	Graft contraction and scar formation are mechanically regulated. Tension, substrate stiffness, wound geometry, and graft–bed adhesion influence fibroblast and myofibroblast activity. Clinical studies seldom quantify mismatch between the substitute and surrounding skin; interface mechanobiology remains largely overlooked.	[58,59,60,73,74,76]
Tissue- engineered vascular grafts	Autologous cell-seeded biodegradable grafts have achieved long-term patency in pediatric low-pressure circulation. Bioengineered vessels have also been evaluated for vascular access and arterial reconstruction.	Stenosis, thrombosis, aneurysmal dilatation, calcification, slow remodeling, limited small-diameter arterial performance, and lengthy or costly production.	Stenosis and tissue overgrowth are repeatedly reported at anastomoses, sometimes around retained sutures. Compliance mismatch, disturbed flow, stress concentration, altered wall strain, and macrophage-mediated remodeling plausibly interact. Surgeons recognize the anastomosis as problematic, but clinically validated graded mechanical transitions remain unavailable.	[80,81,83,84,85,86,91]
Urinary bladder and urethral constructs	Autologous cell-seeded scaffolds have demonstrated feasibility in bladder augmentation and urethral reconstruction, particularly in selected pediatric patients.	Leakage, fistula, fibrosis, contraction, stenosis, insufficient smooth-muscle regeneration, poor vascularization, and inconsistent functional improvement.	The sutured junction must remain watertight during repeated filling–emptying deformation. Abrupt compliance differences, scaffold degradation, suture stress, and micromotion may prevent sealing and promote fibrosis. Clinical reports recognize tension and leakage but rarely establish mechanical discontinuity as an independent cause.	[11,96,99,100]
Tracheal and airway constructs	Segmental allotransplantation and limited patch reconstruction show that airway replacement may be possible under carefully controlled conditions.	Anastomotic fistulae, stenosis, obstructive granulation, infection, graft collapse, inadequate vascularization, absent mucociliary epithelium, and poor integration. Earlier synthetic stem-cell-seeded replacements caused severe complications and major scientific and ethical concerns.	The airway interface experiences cyclic pressure, longitudinal motion, bending, and cartilage-dependent radial support. Stiffness or geometric mismatch can concentrate suture stress; insufficient rigidity promotes collapse. Granulation and fistulae are compatible with mechanically aggravated inflammation, although direct clinical proof is scarce. Surgeons emphasize a tension-free, vascularized anastomosis mainly at a procedural level.	[103,104,105,108,110]
Cardiac TECs	Early collagen–cell patches and recent pluripotent-stem-cell-derived engineered myocardium have demonstrated feasibility, graft vascularization, increased wall thickness, and potential remuscularization.	Arrhythmia, immune rejection, insufficient perfusion, limited electromechanical coupling, delamination, manufacturing complexity, and long-term safety.	The interface must transmit cyclic force, vascularize, and establish electrical continuity. Excessive stiffness may concentrate strain at patch margins; insufficient attachment limits force transfer. Mechanical reinforcement may benefit ventricular geometry even without complete regeneration, but interface strain and coupling are seldom measured clinically.	[113,114,115]
Neural TECs	Nerve conduits and processed allografts are clinically used for selected peripheral-nerve gaps; advanced cell-seeded constructs show strong experimental potential.	Limited regeneration across long gaps, inaccurate fascicular targeting, neuroma, fibrosis, slow reinnervation, and incomplete functional recovery.	Stiffness, topography, alignment, conduit collapse, suture tension, and micromotion influence growth-cone migration and Schwann-cell behavior. Mechanical mismatch may induce fibrosis or glial activation, but clinical nerve studies rarely correlate interface mechanics with regeneration.	[116,117]
Tendon/ ligament and enthesis TECs	Biological patches and scaffolds are used for augmentation; graded and multilayer constructs reproduce aspects of the tendon–bone transition experimentally.	Weak tendon-to-bone integration, scar-mediated healing, tunnel widening, fixation failure, retear, and failure to regenerate the native graded enthesis.	This application provides a particularly clear mechanobiological paradigm: an abrupt soft–hard junction produces stress concentration, whereas the native enthesis distributes load through compositional and stiffness gradients. Loading also regulates stem-cell fate and matrix organization; both overloading and stress deprivation impair integration.	[47,52,119,120]

**Table 2:** Mechanical stimulation paradigms and reported outcomes in engineered soft tissues and translational studies.

Engineered Tissue Model	Type and Parameters of Mechanical Stimulation	Reported Biological or Functional Effects	Relevant Refs.
Skin—fibroblast/keratinocyte skin equivalent on electrospun collagen (in vitro)	Static uniaxial strain: 0%, 5%, 10%, 20%, or 40%; constructs maintained at an air–liquid interface for 10 days.	Intermediate strain was beneficial: 10–20% strain increased tissue strength; 20% increased COL1A1 and fibronectin expression; 5–20% promoted proliferation and epidermal differentiation. Excessive strain did not confer the same benefit.	[164]
Skin—oligomeric collagen dermal-regeneration scaffold in rat full-thickness wounds	Confined compression used to create collagen fibril densities of 4, 20, or 40 mg/cm^3^ and controlled through-thickness density/orientation gradients	Greater fibril density reduced wound contraction and myofibroblast-marker expression. Density and architecture controlled cellular infiltration, vascularization, and epithelial migration.	[75]
Skin—compressed human dermo-epidermal skin substitute (in vitro)	Static circumferential/radial constraint with an anti-shrinkage device during culture	Contraction was limited to approximately 3–8% versus 23–36% in unconstrained controls. Constraint preserved geometry, supported fibroblast morphology and improved epidermal organization.	[77]
Vessel—bovine smooth-muscle-cell/PGA tissue-engineered artery, subsequently endothelialized (preclinical)	Pulsatile radial distension of approximately 5% at 165 beats/min for about 8 weeks.	Conditioning increased collagen accumulation and mechanical strength; burst pressure exceeded 2000 mmHg, suture retention reached approximately 90 g, collagen approached 50% of dry weight, and short-term porcine patency was demonstrated.	[10]
Vessel—fibrin tubular constructs seeded with porcine valve interstitial cells or human dermal fibroblasts	Cyclic distension at 0.5 Hz with a 12.5% duty cycle for 3 weeks. Constant amplitudes of 2.5–20% were compared with an incremental regimen of 5% → 15%.	A constant amplitude near 15% increased ultimate tensile strength by 47%, modulus by 45%, and collagen density by 29% relative to static culture. Incremental loading produced still greater strength/toughness.	[165]
Vessel—hiPSC-derived vascular smooth muscle cells on a 3.2 mm PGA tube	One week static culture followed by 7 weeks of pulsatile radial conditioning at 110–120 beats/min; strain increased from 0.5% to 3% by week 4 and remained at 3% through week 8.	Constructs reached a rupture pressure of 1419 ± 174 mmHg, suture retention of 157.5 ± 16.5 g, and collagen content of 43.1 ± 4.4%, approaching human saphenous-vein performance. Abrupt early loading at about 2% damaged immature constructs.	[166]
Urinary bladder—smooth-muscle/urothelial-cell-seeded porcine acellular matrix or small-intestinal submucosa	Hydrostatic pressure ramped from 0 to 10 cmH_2_O over 55 min, followed by voiding to 0 cmH_2_O over 10 s; cell-seeded scaffolds were cycled for 6 h.	Scaffold integrity and cellular activity were maintained; cells tended to orient perpendicular to the applied pressure. The work established feasibility rather than a mature functional endpoint.	[167]
Urinary bladder—human bladder smooth muscle cells cultured in scaffolds	Cyclic hydrodynamic pressure of 0, 100, 200, or 300 cmH_2_O for 24 h.	Pressures of 200–300 cmH_2_O increased proliferation and activated SGK1; SGK1 silencing abolished the response. These pressures model pathological/supraphysiological loading.	[168]
Urinary bladder—porcine smooth muscle or urothelial cells on type-I collagen	Continuous uniaxial stretch–relaxation at 0.3 µm/s to a maximum strain of about 20% over a 4 h cycle, repeated for 6 days.	Smooth-muscle cells aligned, proliferated, and increased α-SMA and calponin-1 expression. Urothelial cells experienced more construct rupture, showing cell-type-specific mechanical tolerance.	[169]
Trachea—chondrocytes on a PCL/type-II-collagen tubular scaffold	Taylor–Couette continuous flow at 5, 10, 15, or 20 rpm for 4–8 weeks; maximum wall shear approximately 0.189–0.752 dyn/cm^2^ and minimum approximately 0.000303–0.00104 dyn/cm^2^.	Rotation increased proliferation, glycosaminoglycan, and collagen. Cell number doubled, GAG increased about 170%, and collagen about 240% versus static culture. Rabbit implantation showed re-epithelialization but also granulation overgrowth.	[170]
Trachea—chondrocyte-seeded PCL cylinder with muscle-flap prefabrication and reconstruction	Dynamic radial diameter change in a radial-stretch bioreactor; amplitude and frequency were not reported in the abstract.	Mechanical stimulation improved cell infiltration, cartilage-specific gene expression, and extracellular-matrix accumulation, while suppressing fibrous-tissue invasion after reconstruction.	[171]
Airway/lung—decellularized mouse lung seeded with fetal alveolar cells	Room-air ventilation at 180 breaths/min with a tidal volume of 300 µL for 7 days.	The bioreactor preserved extracellular-matrix architecture and supported attachment and maintenance of fetal type-II alveolar epithelial cells.	[172]
Airway/lung—recellularized decellularized rat lung	Physiological vascular perfusion plus negative-pressure liquid/air ventilation for approximately 1 week; detailed waveform magnitude was not reported in the main summary.	Perfusion improved endothelial survival; ventilation improved distal epithelial survival and fluid clearance; air exposure favored type-I alveolar and ciliated phenotypes. Orthotopic grafts exchanged gas for 45–120 min but showed bleeding and reduced inflation.	[173]

## Data Availability

The datasets generated during and/or analyzed during the current study are available from the corresponding author upon reasonable request.
